# Investigation of the phosphorescence, persistent decay and structure properties of Eu^2+^: strontium aluminate doped with Nd^3+^, B^3+^ or Dy^3+^

**DOI:** 10.1186/s13065-025-01651-7

**Published:** 2025-11-15

**Authors:** S. E. Abo Sawan, Y. M. Hamdy, R. M. Khattab

**Affiliations:** 1 Refractories, Ceramics and Building Materials Department, Center of Excellence for Advanced Sciences, National Research Centre, Dokki, Cairo, 12622 Egypt; 2 Spectroscopy Department, National Research Centre, Dokki, Cairo, 12622 Egypt

**Keywords:** Strontium aluminate, Sintering, Active carbon, Decay time

## Abstract

Luminescent sources based on crystalline materials doped with transition metal or rare-earth cations are known as persistent phosphors, and they can continue to emit light even after the excitation source has disappeared. The present study aims to prepare strontium aluminate with varying weight percentages of Eu^2+^, RE^3+^ (RE = Dy^3+^, Nd^3+^, or B^3+^) oxides by the solid-state preparation followed by firing under active carbon at 1250 °C.The produced phosphors were examined using FTIR spectroscopy, mechano-luminescent measurement, scanning electron microscopy (SEM), and X-ray diffraction (XRD). A bulk density and apparent porosity measurement was also used to assess the impact of the firing temperature at 1250 °C. Photoluminescence characteristics were examined concerning the kind of trivalent oxides doped in strontium aluminate. The results showed that for samples containing 0.15 weight% of Eu_2_O_3_ and 0.15 weight% of RE_2_O_3_, SrAl_2_O_4_ formed with Eu^+ 2^, RE^3+^ as a single phase. However, RESr_2_AlO_5_ was formed together with a minor amount of SrAl_2_O_4_ when the Eu_2_O_3_ contents decreased at the expense of the RE_2_O_3_ contents. Additionally, it was observed that the Eu^3+^ was converted into Eu^2+^ for every sample. Every sample had porous behavior, and the bulk density was increased by the inclusion of RESr_2_AlO_5_ in addition to SrAl_2_O_4_. Transitions from the 4f^6^ and 5d^1^ configurations of the emission center (Eu^2+^ ions) to the 4f^7^ configuration result in a broad band at peaks of 517 nm in the emission spectra of all the samples. Samples containing RESr_2_AlO_5_ exhibit high phosphor color characteristics, forming red-orange phosphors encircled by green phosphor rings. Compared to samples containing Nd_2_O_3_ and B_2_O_3_, those containing Dy_2_O_3_ phosphor have the highest decay time values.

## Introduction

Luminescent materials, often known as phosphors, generate light when they absorb energy from an excitation source. Persistent luminescence, also known as afterglow, is an energy-efficient material that releases light for a long time after being stimulated with UV and ionizing radiation has stopped [1–8]. Many afterglow phosphors that produce light in the UV to infrared range have been developed [9]. The finest afterglow phosphors generated in the visible spectrum are aluminates, silicates, or sulfides doped with europium and an appropriate trivalent lanthanide.

Strontium aluminates exhibit remarkable properties, including long decay times, high quantum efficiencies, superior chemical stability, and zero radiation [10]. SrAl_2_O_4_:Eu^2+^, Dy^3+^ is the most commonly seen afterglow phosphor [11]. One helpful technique to alter the phosphor characteristics of phosphorescent materials is to dope them with various ions. Palilla [12] et al. studied SrAl_2_O_3_:Eu^2+^ luminescence in 1968 and, for the first time, observed the lengthy afterglow features of the aluminate system. MAl_2_O_4_: Eu^2 +^ (M = Mg, Ca, Sr, or Ba) is the primary long afterglow photoshor material of the aluminate system, exhibiting superior luminescence performance [12]. Its emission spectra primarily appear in the blue-green light region, and SrAl_2_O_4_:Eu^2+^, Dy^3+^ is the well-known blue-green extended afterglow substance with the best performance [13]. After being excited by ultraviolet or X-ray light, the long afterglow material SrAl_2_O_4_:Eu^2+^, Dy^3+^ can emit a vivid and long-lasting green afterglow. The brightness of the afterglow is about 20 times greater than that of the sulfide structure, the afterglow period exceeds 24 h, and the afterglow emission peak is situated at 520 nm [14]. With the proper dopant selection, SrS, an alkaline earth sulphide (AES) member with a band gap of 4.2 eV, can generally be tailored to distinct emission colors. For instance, the SrS: Eu^2+^ phosphor is an effective deep red phosphor with a cubic crystal structure and a 5d 4f luminous transition in Eu^2+^ that peaks at roughly 620 nm. The Eu^2+^ activator ions are substituted at the host’s Sr^2+^ sites. In the symmetry group Oh, six sulfur anions (S^2−^) encircle each Eu^2+^ ion, resulting in an octahedral shape [15–17]. Aizaw et al. reported on the extended afterglow phosphorescent sensor materials of SrAl_2_O_4_:Eu^2+^ doped with various rare earth ions [18]. They also noted that the thermal excitation of the trapped carrier dominated the long afterglow phosphorescence of SrAl_2_O_4_:Eu^2+^ and that its lifetime and intensity changed along with different doping helpful activators (Y. La, Er, Ce, Nd, Yb, Sm, Gd, Tb, Dy, Pr, Ho, Tm, and Lu). According to Katsumata et al. [19], trap stages in the long-lasting phosphors of SrAl_2_O_4_: Eu^2+^ doped with Nd, Sm, Gd, Dy, and Y were characterized, and the depth and density of the traps had a significant impact on the long-lasting phosphorescence. The phosphorescent properties and mechanism of Eu^3+^ and Cu^2+^ codoped SrAl_2_O_4_ were reported by Yang et al. [20]. Additionally, it was observed that the co-doped phosphors’ excitation and emission spectra differ significantly from those of SrAl_2_O_4_:Eu and SrAl_2_O_4_:Cu. The doping of metal oxide ions could dramatically affect the luminous characteristics of these materials. These long-lasting phosphorescence materials have been prepared using various techniques, including solid-state reaction [21], sol-gel [22], co-precipitation [21], combustion synthesis [23], and reverse microemulsion [24]. These techniques involve firing the prepared materials at temperatures ranging from 1200 to 1700 °C for at least five hours in an atmosphere of reducing gases, such as H_2_-Ar, H_2_-N_2_, H_2_, or NH_3_ [12, 18–24].

The use of active carbon as a reducing environment to convert Eu^3+^ to Eu^2+^ has received little attention. Using a home microwave oven set to 2.45 GHz and 500 W for 30 min, Tadashi Ishigaki et al. [25] produced SrAl_2_O_4_: Eu^2+^, Dy^3+^ phosphor by carbon reduction processes. They discovered that heating with carbon produced a reductive environment and heating energy, making it an effective microwave absorber.

According to A. Nor Nazida et al. [26] found that single firing in a graphite crucible at a lower firing temperature improves the luminous qualities. To lower the temperature and save money and time, a graphite crucible was used to create a reducing environment.

The improved characteristics were effectively produced by solid-state reaction. The optimum firing temperature was considerably decreased to 1250 °C. The results confirmed the presence of a monoclinic structure of SrAl_2_O_4_ as a main phase and the intermediate phase characterized as an orthorhombic structure of Sr_4_Al_14_O_25_. It was determined that the combination of 0.5 mol % Eu_2_O_3_ and 1 mol % Dy_2_O_3_ is the best for the high photoluminescence effect in this system.

Conversely, in our research, we focus on preparing these phosphor materials by dipping them into active carbon and then firing the obtained samples in furnaces with an oxygen-rich environment to substitute for the gas-reducing atmosphere [27], which lowers the cost and energy consumption of the end product. We prepared different host lattices containing Eu^2+^ with Dy^3+^, Nd^3+,^ or B^3+^ ions (as activators) with various concentrations to ascertain how the characteristics of photoluminescence materials are affected by the increase in tri-metal oxide and the shift in the atmosphere to active carbon. Additionally, creating materials that emit light continuously for as long as possible can be used in many application areas, including oxygen sensors, Glow watch dial pads, radiation dosimetry, lighting apparatus, switches, luminous paint, electronic instrument dial pads, escape routes, warning signs, and light-emitting diode luminous paints in highways and airports, as well as writing and printing inks, panels, and plasma display phosphors [28, 29].

## Materials and methods

Strontium carbonate SrCO_3_ (MW = 147.64) was purchased from the British Drug House LTD, and Aluminum oxide (Al_2_O_3_, with purity 99%) was given by the Alexandria company. Trivalent Europium (III) oxide Eu_2_O_3_ (99.9%) and dysprosium (III) oxide Dy_2_O_3_ (99.9%) were purchased from Alfa Esar. Boron Oxide, 98%, -40 mesh from Acros Organics (MW = 69.61 and d = 2.460).

**Fig. 1:**
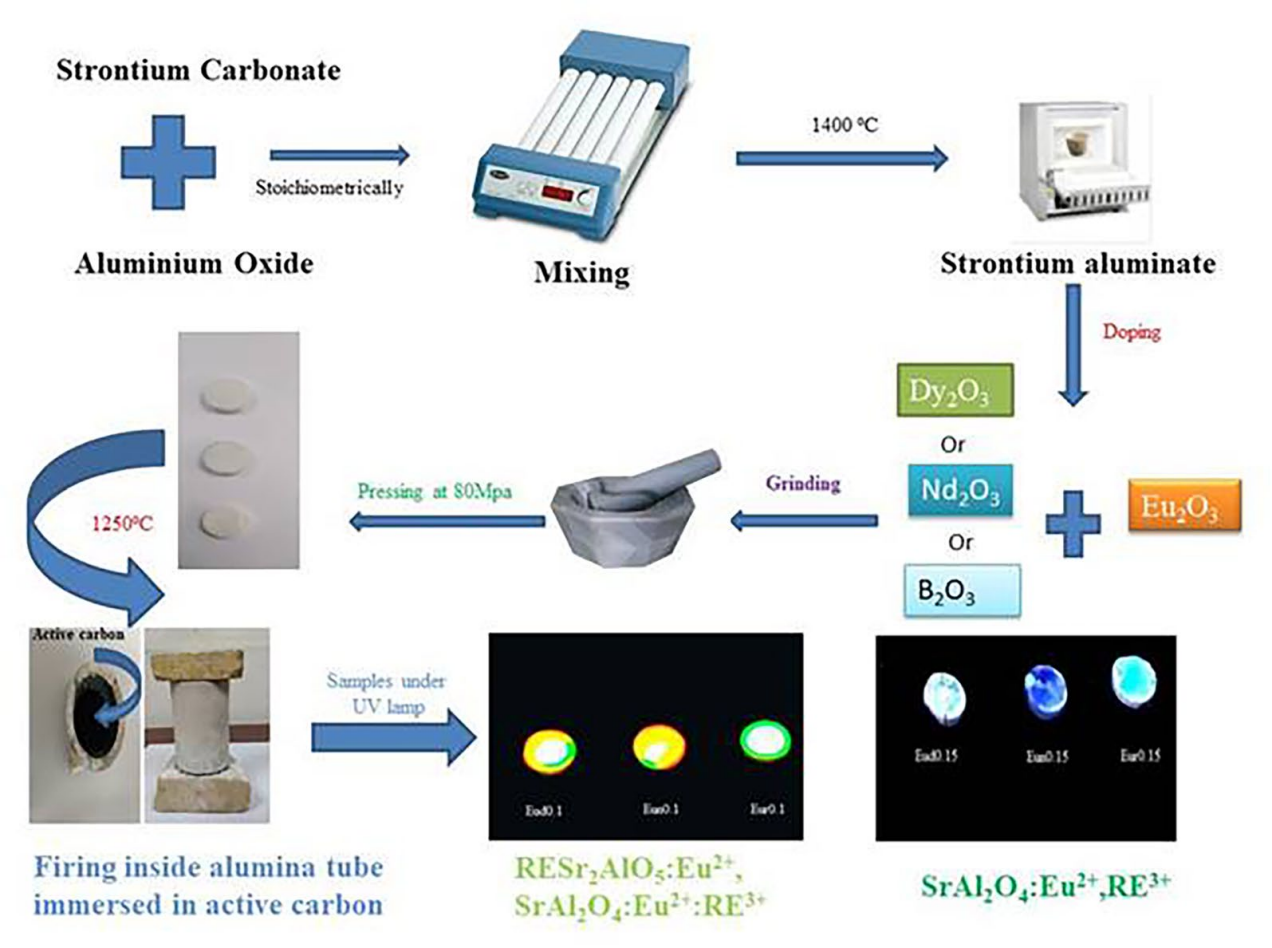
Schematic representation for preparation of the phosphor strontium aluminate

Figure 1 shows a schematic representation of the phosphor strontium aluminate preparation process. Strontium aluminate (SrAl_2_O_4_) powder was first produced stoichiometrically [27] and fired at 1400 °C for one hour of soaking time at a rate of 10 °C/h. This step is crucial for preparing the phosphor materials’ host lattice and guaranteeing that SrAl_2_O_4_ crystallization occurs.

After the addition of rare earth occurred, this step is carried out as follows: several trivalent oxides (Eu_2_O_3_ with Dy_2_O_3_, Nd_2_O_3_, or B_2_O_3_) were combined with the produced strontium aluminate powders with varying weight ratios (0.3 weight% based on powder) as shown in Table 1. The solutions were agitated for four hours in plastic jars containing acetone and alumina balls. The samples were dried for 24 h at 60 °C. After drying, the powders were ground in a porcelain mortar to break up accumulations. Afterwards, uniaxial pressing at 30 MPa was used to create disk pellets [22, 28, 29]. To convert Eu^3+^ to Eu^2+^ and prevent it from oxidizing again, especially at low temperatures, active carbon powder is introduced into an alumina tube and then fired under rapid firing conditions. Because the body has been prepared in pellet form, the unreacted powder from the first stage is finished in this phase. In an alumina tube sealed on both sides with refractory breaks, the samples were then immersed in active carbon. The tube was then placed in a furnace and fired at 1250 °C for 60 min at a rate of 30 °C per minute. Rapid firing is required to stop carbon from oxidizing to carbon dioxide while also accelerating the rate at which Eu^3+^ is reduced to Eu^2+^. After that, the tube’s active carbon is extracted, and the furnace is cooled.

**Table 1:** Composition of phosphors strontium aluminate

Sample name	SrAl_2_O_4_ Prepared Powder calcined at 1400 °C	Eu_2_O_3_	Dy_2_O_3_	Nd_2_O_3_	B_2_O_3_
	wt%				
Eud0.1	99.97	0.1	0.2	–	–
Eud0.15	99.97	0.15	0.15	–	–
Eun0.1	99.97	0.1	–	0.2	–
Eun0.15	99.97	0.15	–	0.15	–
Eur0.1	99.97	0.1	–	–	0.2
Eur0.15	99.97	0.15	–	–	0.15

### Characterization

The samples were analyzed using Bruker D8 Advance-Germany, Target Cu Kα, with a secondary monochromator, V = 40 kv, A = 40 mA, and a Ni filter to determine their crystalline structure, size, and phase composition. A JASCO FT/IR-6100 recorded FTIR spectra of produced samples. At 25 ^◦^C, the spectrum was captured at a resolution of 4 cm^− 1^, spanning from 400 to 4000 cm^− 1^. The Archimedes method calculates the bulk density and apparent porosity of the burnt samples. A hydraulic testing apparatus measured compressive strength (SEIDNER, 794).

Following coating with thin gold films, the morphology of the produced samples was examined using a Scanning Electron Microscope (SEM) model FEI, Quanta FEG, 250.

Photoluminescence spectra, comprising emission, excitation, phosphorescence, and afterglow decay curves, were ascertained through the use of a spectrofluorometer (Jasco FP-6500, Japan) that was outfitted with a 150-watt Xenon arc lamp for illumination. All the samples were measured at room temperature and under the same geometrical conditions for comparison. The samples were irradiated by UV light of the spectrofluorometer at 365 nm for 10 min before decay curve measurements.

## Results and discussions

### Phase composition of the synthesized phosphors

**Fig. 2:**
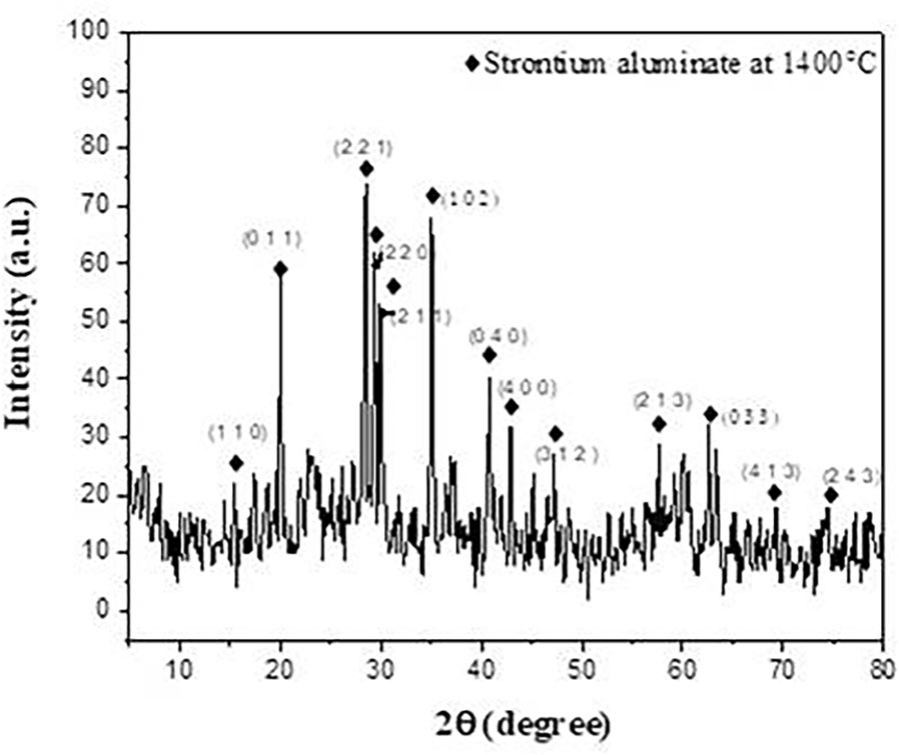
XRD patterns of SrAl_2_O_4_ fired at 1400℃

Figure 2 displays a typical X-ray diffraction pattern of the SrAl_2_O_4_ powders produced after being synthesized at 1400 °C. The produced powder’s XRD patterns were measured and contrasted with monoclinic SrAl_2_O_4_ PDF standard cards (No. 74–0794).

As can be observed, the XRD patterns mostly show the formation of monoclinic phase diffraction peaks of SrAl_2_O_4_, which is consistent with the earlier findings [30, 31], as depicted in Fig. 2. Furthermore, certain noise peaks may have formed as a result of the particles’ rapid heating and the great distance between them that decreases the rate of the reaction to occur. Later, in the second firing state, this unreacted phase is finished.

The interfacial interaction between SrCO_3_ and α-Al_2_O_3_ is responsible for the solid-state production of SrAl_2_O_4_ and occurs as follows:-.

At 1000 °C, SrO combines with Al_2_O_3_ to form SrAl_2_O_4_ in a locally reached 1:1 M stoichiometric ratio. This reaction is caused by strontium carbonate breaking down into SrO, which is shown as follows:

1 $${\text{SrO}}\,+\,{\text{C}}{{\text{O}}_{\text{2}}}\,=\,{\text{SrC}}{{\text{O}}_{\text{3}}}$$

2 $${\text{SrO}}\,+\,{\text{A}}{{\text{l}}_{\text{2}}}{{\text{O}}_{\text{3}}}\,=\,{\text{SrA}}{{\text{l}}_{\text{2}}}{{\text{O}}_{\text{4}}}$$

Conversely, SrAl_4_O_7_ was produced at temperatures more than 1000 °C, and this is ascribed to the chemical interaction between SrO and Al_2_O_3_ as a result of a locally developed 1:2 M stoichiometric relationship, as demonstrated by the following reaction:

3 $${\text{SrO}}~+~{\text{2A}}{{\text{l}}_{\text{2}}}{{\text{O}}_{\text{3}}}\, \to \,{\text{SrA}}{{\text{l}}_{\text{4}}}{{\text{O}}_{\text{7}}}$$

4 $${\text{SrA}}{{\text{l}}_{\text{2}}}{{\text{O}}_{\text{4}}}\,+\,{\text{A}}{{\text{l}}_{\text{2}}}{{\text{O}}_{\text{3}}}\, \to \,{\text{SrA}}{{\text{l}}_{\text{4}}}{{\text{O}}_{\text{7}}}$$

Nevertheless, this phase breaks down as follows, since it is not stable at this temperature:

At temperatures more than 1150 °C, the leftover SrO combines with Al_2_O_3_ to generate Sr_3_Al_2_O_6_, as demonstrated by the following reaction:

5 $${\text{3SrO}}\,+\,{\text{A}}{{\text{l}}_{\text{2}}}{{\text{O}}_{\text{3}}} \to {\text{S}}{{\text{r}}_{\text{3}}}{\text{A}}{{\text{l}}_{\text{2}}}{{\text{O}}_{\text{6}}}$$

6 $${\text{S}}{{\text{r}}_{\text{3}}}{\text{A}}{{\text{l}}_{\text{2}}}{{\text{O}}_{\text{6}}}\,+\,{\text{2A}}{{\text{l}}_{\text{2}}}{{\text{O}}_{\text{3}}} \to {\text{3SrA}}{{\text{l}}_{\text{2}}}{{\text{O}}_{\text{4}}}$$

By raising the temperature above 1200 °C, Sr_3_Al_2_O_6_ changes into SrAl_2_O_4_ [32–34].

**Fig. 3:**
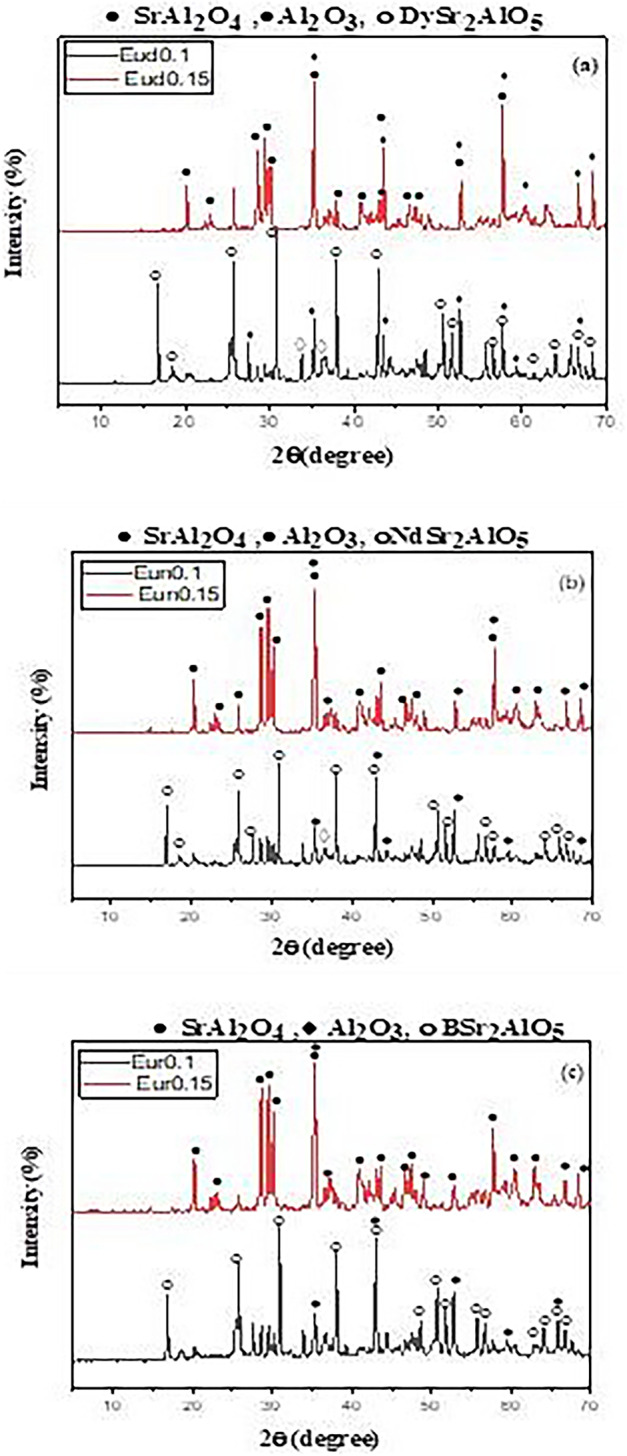
The XRD pattern of the prepared strontium aluminate samples fired under active carbon at 1250℃ in presence of different trivalent oxides

In the XRD patterns (Fig. 3) for the Eud0.15, Eun0.15, and Eur0.15 samples, pure monoclinic phase diffraction peaks of SrAl_2_O_4_ predominate, consistent with the earlier observations. The absence of any other product or beginning material indicates that the precursor powders have a phase composition of low-temperature monoclinic phase (α-phase). As seen from Fig. 3, incorporating Nd^3+^ and B^3+^ ions enhances the formation of the SrAl_2_O_4_ phase rather than the Dy^**3+**^ ion.

Therefore, the addition of rare earth ions in high percentages did not affect the host’s crystal structure and agglomerated. Rietveld refinement investigations [35] indicate that rare earth ions can substitute at all cationic sites when integrated into the crystal structure of SrAl_2_O_4_. The ionic radii of Al^3+^ 0.053 nm and Nd^3+^ (0.0983 nm cannot be replaced by Eu^2+^ (0.117 nm) due to the mismatch between each ionic radius. Eu^2+^/Nd^3+^ ions favor occupying Sr^2+^ sites (Sr^2+^=0.127 nm) [36]. SrAl_2_O_4_ is enhanced by Nd^3+^ ion addition rather than Dy^3+^ addition because Sr^2+^ and Nd^3+^ have ionic radii that are more similar than Dy^3+^ (Dy^3+^=0.091 nm) [37].

Songhak Yoon [38] explained the enhanced SrAl_2_O_4_ phase formation in the case of B_2_O_3_ introduction. He claimed that the B^3+^ ion caused the SrAl_2_O_4_ crystal lattice to shrink, resulting in a greater number of crystal defects, which is what allowed for the creation of SrA_l2_O_4_ hosts. Shanshan Yea [39] demonstrated a similar outcome, showing that B_2_O_3_ up to 2.5 mol% promotes the crystallization of SrAl_2_O_4_. This is because B^3+^ is much smaller than Al^3+^, which causes the lattice to compress and form additional flaws when B_2_O_3_ is added.

The decrease in the intensity of SrAl_2_O_4_ phases for patterns Eun0.1, Eud0.1, and Eur0.1 prepared with a low concentration of Eu rare earth element was observed in Fig. 3. A new phase called RESr_2_AlO_5_ (R = Nd^3+^, Dy^3+^or B^3+^ions) developed with small amounts of SrAl_2_O_4_ and aluminum oxide as the rare earth act as a dopant. The XRD pattern of RESr_2_AlO_5_ is similar to the EuSr_2_AlO_5_ XRD card (PDF 70-2197) [40]. The best intensity of RESr_2_AlO_5_ was observed for the sample containing Dy_2_O_3_ rather than Nd_2_O_3_ and B_2_O_3_.

Thus, the doping of calcined powder with a low amount of trimetal oxide (Dy_2_O_3_, Nd_2_O_3_, or B_2_O_3_) enhances the formation of SrAl_2_O_4_, as seen in Fig. 3, as its small amount is not sufficient to complete the reaction to form the luminescent structure RESr_2_AlO_5_. While in the case of a high concentration of trimetal oxide at the expense of Eu_2_O_3_ (Eud0.1, Eud0.1, and Eur0.1) and the presence of the weak reducing atmosphere, the reaction entirely occurred by forming the luminescent structure RESr2AlO5: Eu2+ (RE = Dy^3+^, Nd^3+^, B^3+^) beside SrAl_2_O_4_: Eu^2+^, RE^3+^.

Similar to this rapid formation of RESr_2_AlO_5_ with SrAl_2_O_4_, Wanping Chen et al. [41] used a high-temperature solid-state technique to manufacture La_0.97_Sr_2−x_Ca_x_AlO_5_:0.03Eu. It was shown that when x = 1 is adjusted to x = 1.5, the product phase rapidly changes from La(Ca, Sr)AlO_4_ to LaSr_2_AlO_5_. Furthermore, P. J. Chaware [42] discovered that when oxidation occurred in an oxygen atmosphere, a comparable peak of SrAl_2_O_4_ formed with other peaks. This rapid change in our work could perhaps be due to the use of a weak reducing environment and the presence of SrO_2_ and Al_2_O_3_ in an amorphous phase that reacted with excess trimetal oxide (Dy_2_O_3_, Nd_2_O_3,_ and B_2_O_3_) and formed the RESr_2_AlO_5_ compound with SrAl_2_O_4_.

### FT- infrared measurements

**Fig. 4:**
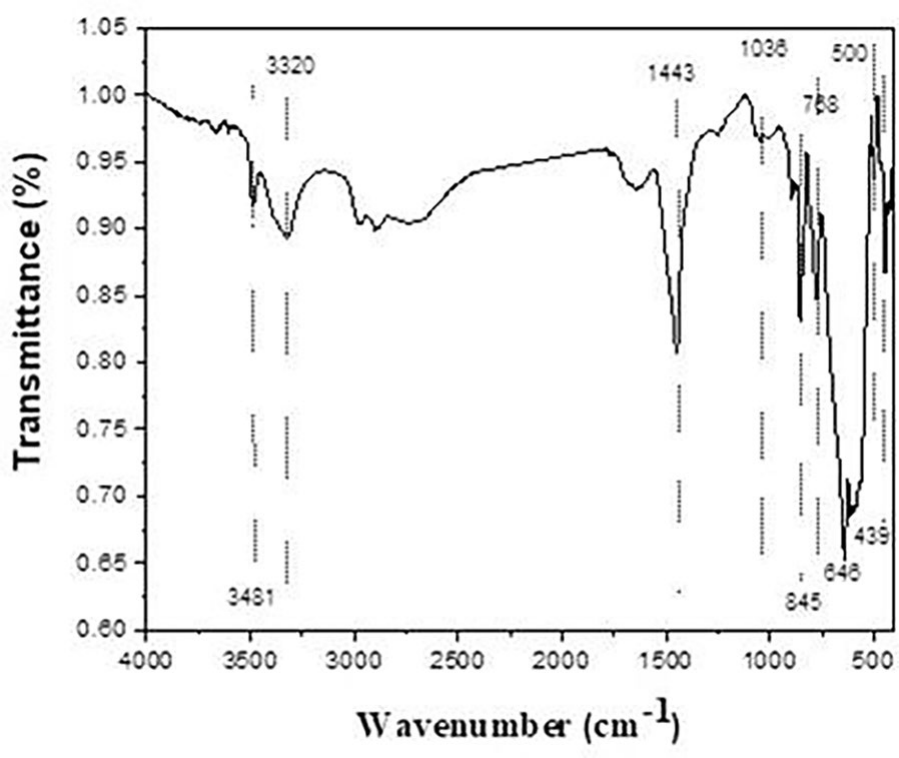
IR spectra of the prepared strontium aluminate fired at 1400℃

The infrared transmission spectra of prepared phosphors fired at 1400˚C are shown in Fig. 4. The Fourier conversion Infrared spectra were captured within the 400–4000 cm^− 1^ wave number range. A series of absorption peaks in the 400–900 cm^− 1^ range revealed the monoclinic crystal structure of SrAl_2_O_4_. Al–O, Sr–O, and Sr–O-Al bond vibrations are linked to the metal-oxygen stretching frequencies in Fig. 4 that vary from 400 to 1000 cm^− 1^. Attributing the antisymmetric stretching bonds at 500 and 646 cm^− 1^ to the Sr-O vibrations is possible. The symmetric bonding of O-Al-O is most likely the cause of a band at 439 cm^− 1^ [43]. The high peak of the sample at 845 cm^− 1^ was linked to the formation of SrAl_2_O_4_ [44]. The absorption bands arise from the vibrations in CO_3_^− 2^ between 400 and 1800 cm^− 1^, which in turn contribute to the vibrations of SrCO_3_ at 1443, 845, and 768 cm^− 1^, respectively, resulting in asymmetric and symmetric C–O stretching, out-of-plane bending, and in-plane bending. Furthermore, at 493 and 500 cm^− 1^, stretching vibrations of the distinctive Al–O and Al–O–Al bond peaks of Al_2_O_3_ are observed [45]. Broadband is seen in the spectra between 3320 cm^− 1^ and 3481 cm^− 1^ due to the free and hydrogen-bonded hydroxyl groups (OH^−^stretching vibrations).

**Fig. 5:**
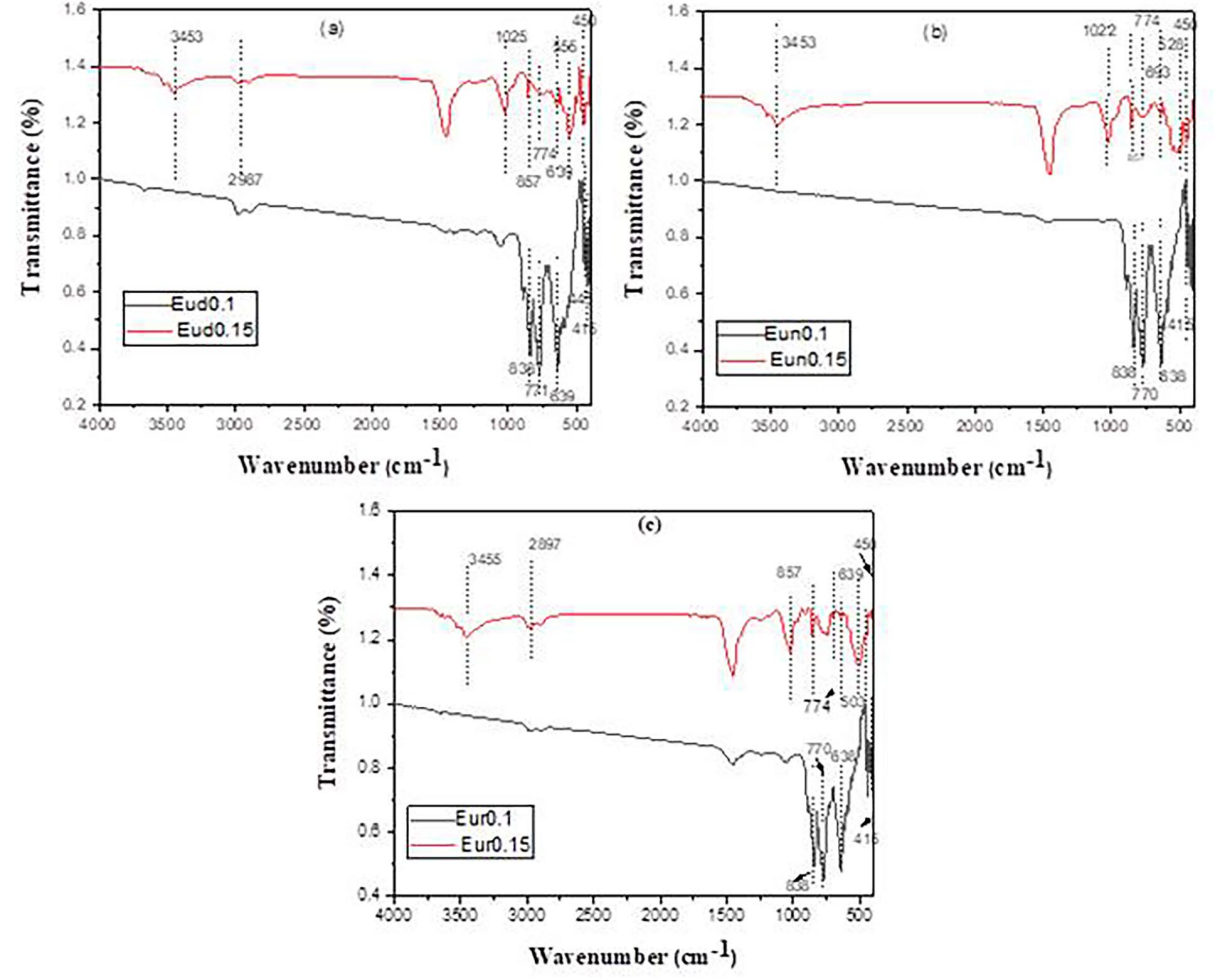
IR pattern of the prepared strontium aluminate samples fired under active carbon at 1250℃ in presence of different axides

As shown in Fig. 5, the spectra of Eud0.15, Eun0.15, and Eur0.15 samples are similar in peaks with different intensities. The peak around 845 cm^-1^ [43, 46] still exists due to an excess of SrAl_2_O_4_ (which is confirmed with XRD). Al–O and Sr–O–Al bond vibrations are linked to metal oxygen stretching frequencies near 700–1000 cm^–1^ [43].

The spectra of the Eud0.1, Eun0.1, and Eur0.1 samples (Fig. 4) show an intense vibration at around 1450 cm^− 1^ [47], which can be attributed to the stretching vibration of tetrahedral AlO_4_. This indicates that SrAl_2_O_4_ remains present in significant amounts, as observed and discussed in the XRD analysis. In addition, distinct peaks are observed between wavenumbers 1000 cm^− 1^ and 400 cm^− 1^, indicating the presence of metal-oxygen bonds. SrO at approximately 639 cm^− 1^, Al–O between 774 and 857 cm^–1^, and Al–O at 450 cm^–1^ are the representative values for the symmetric vibration of O–Al–O [48].

### Microstructure of the prepared luminescent materials:-

**Fig. 6:**
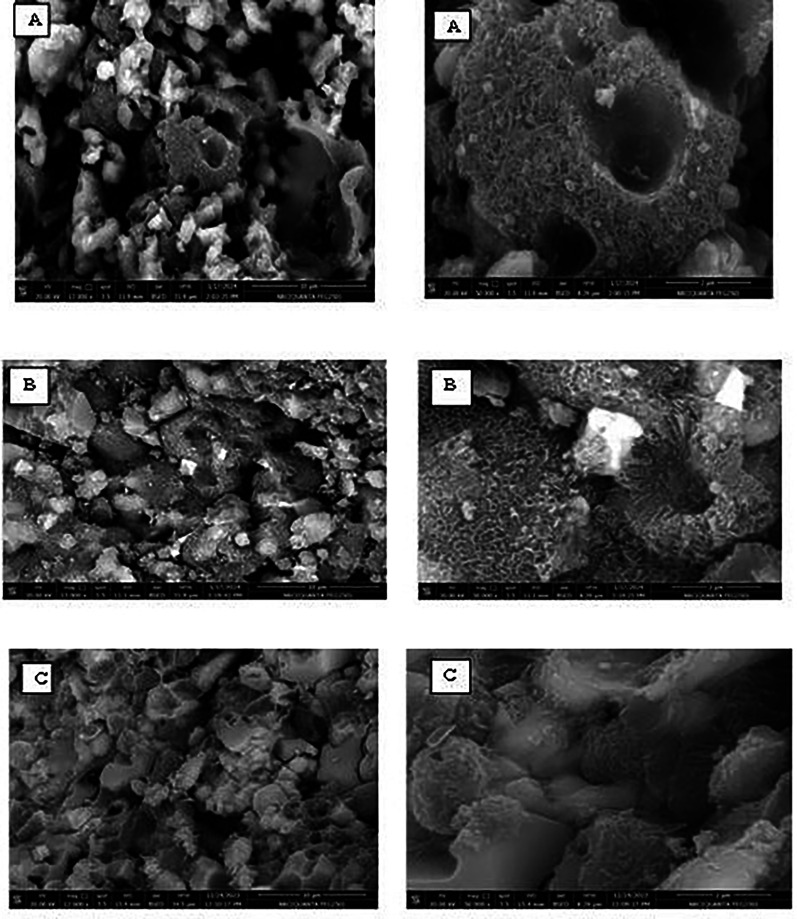
SEM of prepared phosphors (**A**) Eud0.1, (**B**) Eun0.1 and (**C**) Eur0.1 samples

Figure 6 shows the SEM micrographs for samples Eud0.1, Eun0.1, and Eur0.1 at different magnifications. The majority of them are different-sized faceted crystals.

The powders were in the shape of clusters, which resembled coral structures, as seen in Fig. 6 (Eud0.1) and (Eun0.1). It was observed that powders were all comparable to coral structures. Furthermore, a portion of the needle-like bulk phosphor started to stick to the large-grained bulk phosphor’s groove surface [49]. It was discovered that the small-sized, needle-shaped phosphors had essentially covered the surface of the large-particle pieces. Subsequently, the needle phosphors seemed to aggregate on the block phosphor surface, forming cluster needle formations with a uniform covering [49]. This needle shape refers to SrAl_2_O_4_ phosphor.

**Fig. 7:**
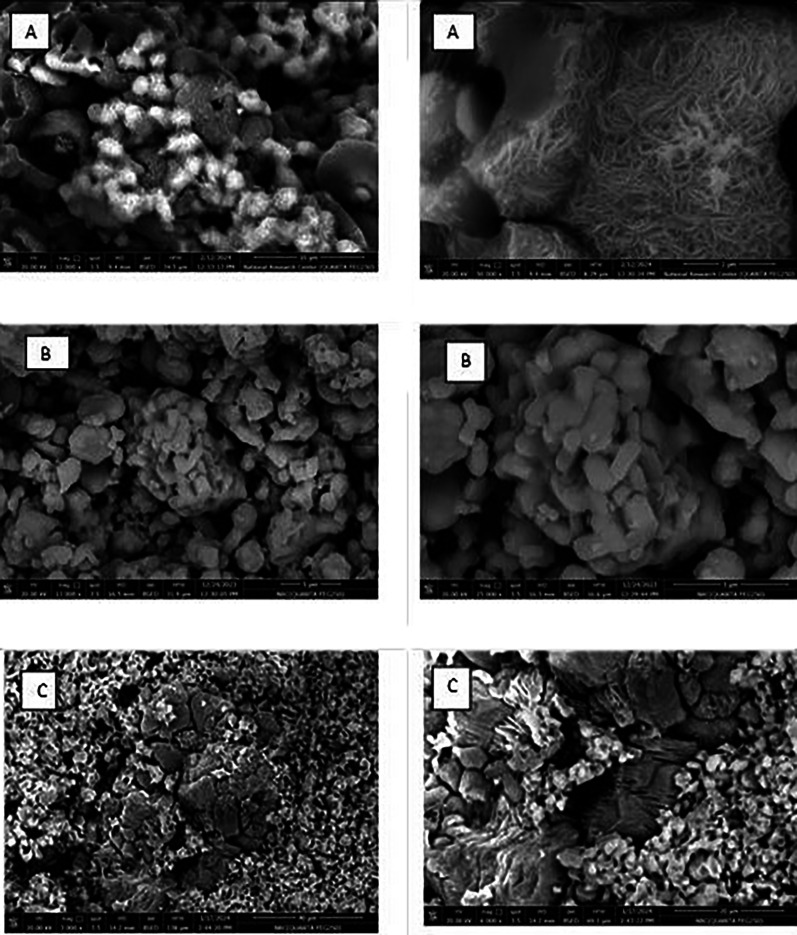
SEM of prepared phosphors (**A**) Eud0.15 (**B**) Eun0.15 and (**C**) Eur0.15 samples

SEM examinations examined changes in the prepared phosphors (Eud0.15, Eun0.15, and Eur0.15). As shown in Fig. 7, some irregular particles appeared, along with some spherical and needle particles that belong to the formation of SrAl_2_O_4_ [50]. The needle particles surround the irregular particles in Fig. 7a, which belong to the SrAl_2_O_4_ phosphor.

That is confirmed by the photograph image of samples in Fig. 1, i.e., reddish-orange phosphorescence encircled by a green ring. The corresponding high-magnification SEM image reveals that the B_2_O_3_-containing sample has many tiny flakes [21]. These small plates overlapped with one another, creating compact sheet structures. When B_2_O_3_ is added, the particles’ morphology becomes more asymmetrical and has faceted edges.

### Physical properties

**Fig. 8:**
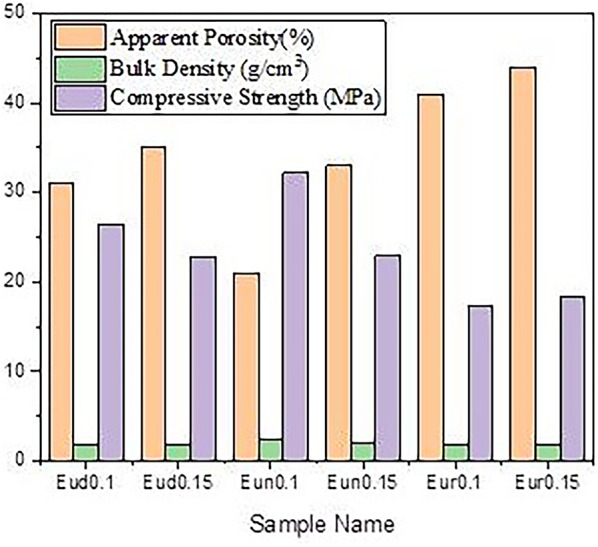
Apparent porosity, Bulk density and compressive strength of prepared phosphors samples

Figure 8 displays the bulk density and apparent porosity of the fired-prepared samples. It was observed that the type of activator (Dy, Nd, or B elements) with the primary activator (Eu) significantly affected the apparent porosity and the bulk density differently as they were added in different weight ratios.

The apparent porosity of Eud0.1, Eun0.1, and Eur0.1 samples is smaller than that of Eud0.15, Eun0.15, and Eur0.15 samples. This results from the creation of some Al_2_O_3_ and SrAl_2_O_4_ between the RESr_2_AlO_5_ grain boundaries, which fill part of the pore regions.

Furthermore, a different reason why these might be less porous could be due to the difference in the crystal structure between RESr_2_AlO_5_ (RE: Dy, Nd, B) and the SrAl_2_O_4_ phase. This can be described as follows: although the crystal structure is similar to the spinel structure of AB_2_O_4_, strontium aluminate, or SrAl_2_O_4_, has a stuffed tridymite-type structure. The large size of Sr^2+^ (1.18 Å) may cause this behavior as it inhibits the formation of the spinel structure [51, 52].

Two crystallographic polymorphs exist for strontium aluminate, or SrAl_2_O_4_: hexagonal symmetry, the ideal, undistorted structure, which is stable above 650 °C, and monoclinic symmetry, which is stable below this temperature. Several authors prove that the transformation from the monoclinic phase (P21) to the hexagonal phase (P6_3_22) is a reversible process [53]. To address the discrepancies in the data, Avdee et al. [54] conducted a thorough analysis of the structural behavior. They postulated a second-order transition to P6_3_22 at 860 °C and a monoclinic → hexagonal phase transition at 680 °C, corresponding to the space group P6_3_.

SrAl_2_O_5_ has a tetragonal structure with eight crystallographic sites. This structure reveals details about the doping of incoming activator ions and possibly the luminescent center of the host matrix. The three types of polyhedra that collectively make up the RESr_2_AlO_5_ crystalline structure are [R/SrO_8_, [SrO_10_], and [AlO_4_] units [55]. In this structure, the host R^3+^ ions are found at half of the 8 h crystallographic sites, generating an eight-fold coordination polyhedral unit known as [R(1)O_8_]. While Sr (3) occupies 4a positions and forms [Sr(3)O_10_] coordinated polyhedra, Sr(2) fills the other half of the 8 h atomic sites and forms [Sr(2)O_8_] polyhedra.

Similarly, Sr^2+^ has two distinct locations. In addition, four oxygen atoms are coordinated with Al to form the Al (4)O_4_ unit found in the 4b crystal locations. The O (5) and O(6) occupy the remaining 4c and 16 l positions, respectively [55].

Thus, the high porosity of Eud0.15, Eun0.15, andEur0.15 is attributed to SrAl_2_O_4_ having a hexagonal structure with low active sides, where (Eud0.1, Eun0.1, and Eur0.1 samples) exist in tetragonal structure with 8 crystallographic site in their structure (DySrAl_2_O_5_), that is significantly affected in coordination of their structure and subsequently on their porosity.

It has been discovered that samples containing Dy_2_O_3_ or Nd_2_O_3_ are less porous than those containing B_2_O_3_. This can be attributed to the radius of the element that fills pores. Dy^+ 3^ and Nd^+ 3^ cations have 0.0912 nm and 0.0983 nm, respectively, where B^+ 3^ has a smaller atomic radius equal to 0.001 nm [56], forming a more porous structure in the samples.

### Compressive strength

The measured compressive strength of the fired sample is displayed in Fig. 8, and the result is consistent with the apparent porosity measurement. All the samples show low strength values. However, sample C, which has the lowest porosity and is doped with boron, has a higher result for compressive strength. For porous materials, an increase in total porosity generally decreases compressive strength [57]. This tendency is nearly universal. Previous research suggests that variations in the strength of the porous body caused by the growth of the grains increased the number of contacting sites inside the porous body. It takes center stage as the primary cause of the porosity shift. Furthermore, several academic publications [58, 59] claim that pores degrade mechanical properties. Because more significant defects cause more fractures in ceramic components than smaller ones, porous ceramics with more prominent pores are expected to have lower mechanical strengths than those with smaller pores, even in cases where porosity is the same.

### Photographs of the samples

An LED chip-phosphor system can produce white light; various phosphorescence behaviors are seen when samples are exposed to LEDs. Samples Eud0.1, Eun0.1 and Eur0.1 showed reddish-orange phosphorescence behaviors encircled by a green ring, as shown in Fig. 1. This is because visible spectrum colors like red, green, and yellow are reemitted by the phosphor, which substantially absorbs ultraviolet (UV)-blue light (370–460 nm) from LED chips. Hence, the red-orange phosphor is associated with the existence of RESr_2_AlO_5_ and is surrounded by SrAl_2_O_4_: Eu^2+^, R^3+^ (R^3+^=Dy^3+^, Nd^3+^, or B^3+^) [60].

Regarding samples Eud0.15, Eun0.15, and Eur0.15, following exposure to LED, these samples exhibit blue-green phosphorescence behaviors (Fig. 1) associated with the production of SrAl_2_O_4_:Eu^2+^,R^3+^ phosphor [61].

### Photoluminescence excitation and emission spectra:-

Photoluminescence spectroscopy, including fluorescence and phosphorescence, deals with the ability of the material to absorb and emit light due to electronic transitions. However, the emission process in phosphorescence involves intersystem crossing from excited singlet states to triplet states, followed by radiative relaxation to the ground state. While in fluorescence, radiative relaxation occurs directly from excited singlet states to the relevant ground states. Therefore, the phosphorescence process takes more time (10 ^–6^ s to seconds) than that of the fluorescence process (10^ − 9^ to 10^ –6^ s). While fluorescence measurements reveal the main electronic transition, which is responsible for the emission of light, phosphorescence measurements show the material’s ability to store light energy. In the case of studying afterglow materials, both fluorescence and phosphorescence measurements are essential to understand the light emission process. Typically, phosphorescence spectra are measured with a delay time (in milliseconds) to distinguish them from fluorescence spectra and obtain pure phosphorescence spectra.

**Fig. 9:**
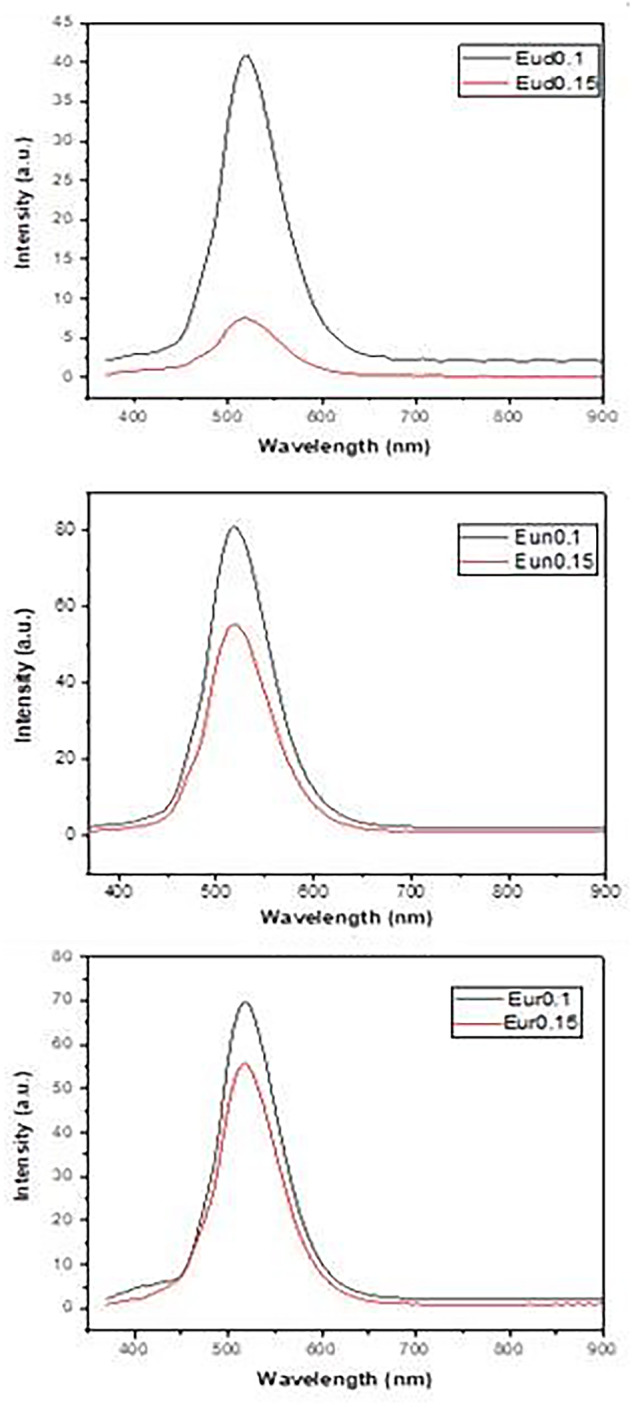
Emission spectra of the prepared samples

Figure 9 displays the fluorescence emission spectra of the prepared samples. For all samples, the emission spectra exhibit a broad band peaking at approximately 517 nm at the excitation wavelength of 365 nm. It is observed that the emission intensity of samples Eud0.1, Eun0.1, and Eur0.1 is higher than that of Eud0.15, Eun0.15, and Eur0.15. This may be due to the crystallization of SrAl_2_O_4,_ with RESr_2_AlO_5_ as was indicated by XRD Fig. 2. As seen previously in the photograp images (Fig. 1), the complete green ring (Eu^2+^, B^3+^:SrAl_2_O_4_) is formed in this sample that surrounds the red-orange phosphor (BSrAl_2_O_5_).

**Fig. 10:**
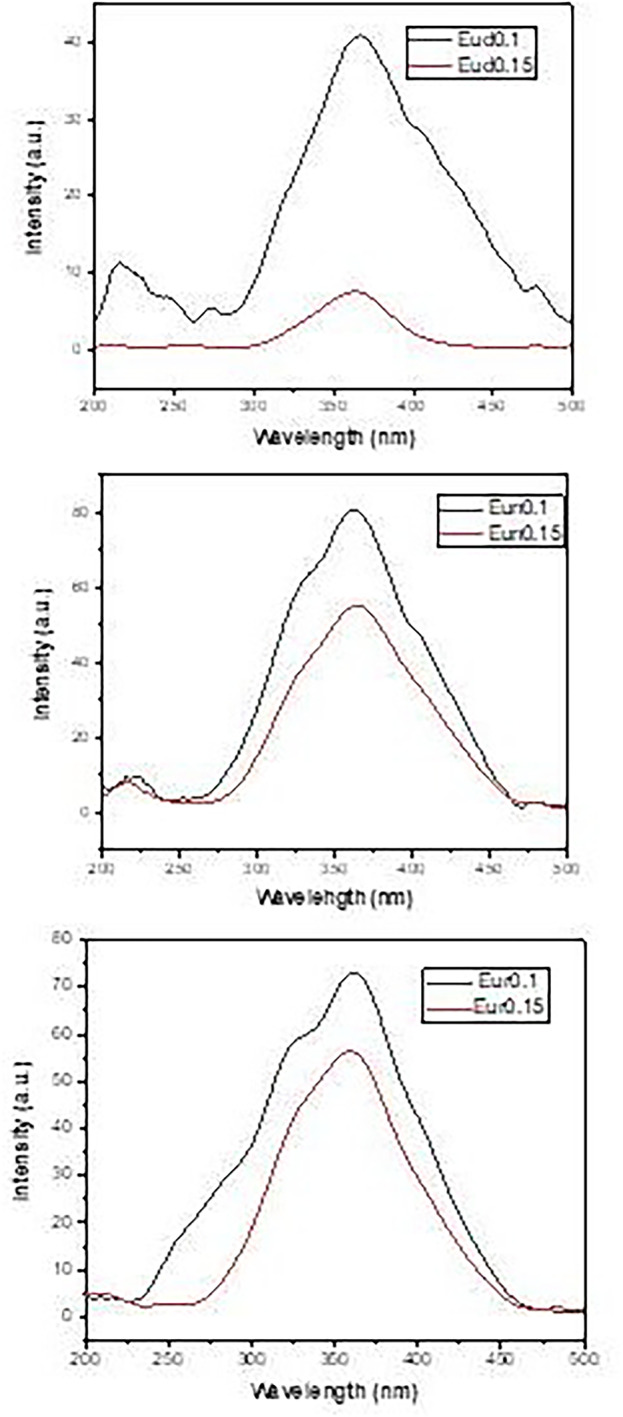
Excitation spectra opf the prepared samples

The excitation spectra in Fig. 10 exhibited the same manner as in the emission spectra, with a broad band peaking at about 365 nm. The excitation band is due to the electronic transition of Eu^2+^ ions 4f ^7^→4f^6^5d^1^ as a result of the absorption of UV light at 365 nm [23, 62].

In all samples, Eu^2+^ ions are the luminescent emitters due to the 4f^6^ → 5d^1^4f^7^ transition corresponding to Eu^2+^ ions and resulting in its green emitted color. The holes in the traps caused by the stimulation of UV light produced a lengthy afterglow. Holes are created from the 4f level to the valence band, and the electrons of Eu^2+^ ions in the 4f level are moved to the 5d level. A portion of the lattice’s open holes is carried to the conduction band and ensnared by dopants Dy^3+^, Nd^3+^, or B^3+^ hole traps. When UV radiation stops, some of the holes that the activator ions have taken from the valence ions are thermally released into the valence band and revert to the ground state of the Eu^2+^ ions, which causes the emission of green light. Consequently, by creating deep hole traps that lengthen the afterglow time, the Dy^3+^, Nd^3+^, or B^3+^ contribute significantly to the long afterglow process.

Substitution of Nd^3+^ or B^3+^ ions for Dy^3+^ ions caused no change in the shape of the emission peaks or their locations. Also, there was no change in the emission and excitation spectra of all the samples. The observed differences were the changes in luminescence intensity and the decay time of the afterglow curves, which indicate that the type of Dy^3+^, Nd^3+^, or B^3+^ ions is responsible for these changes.

**Fig. 11:**
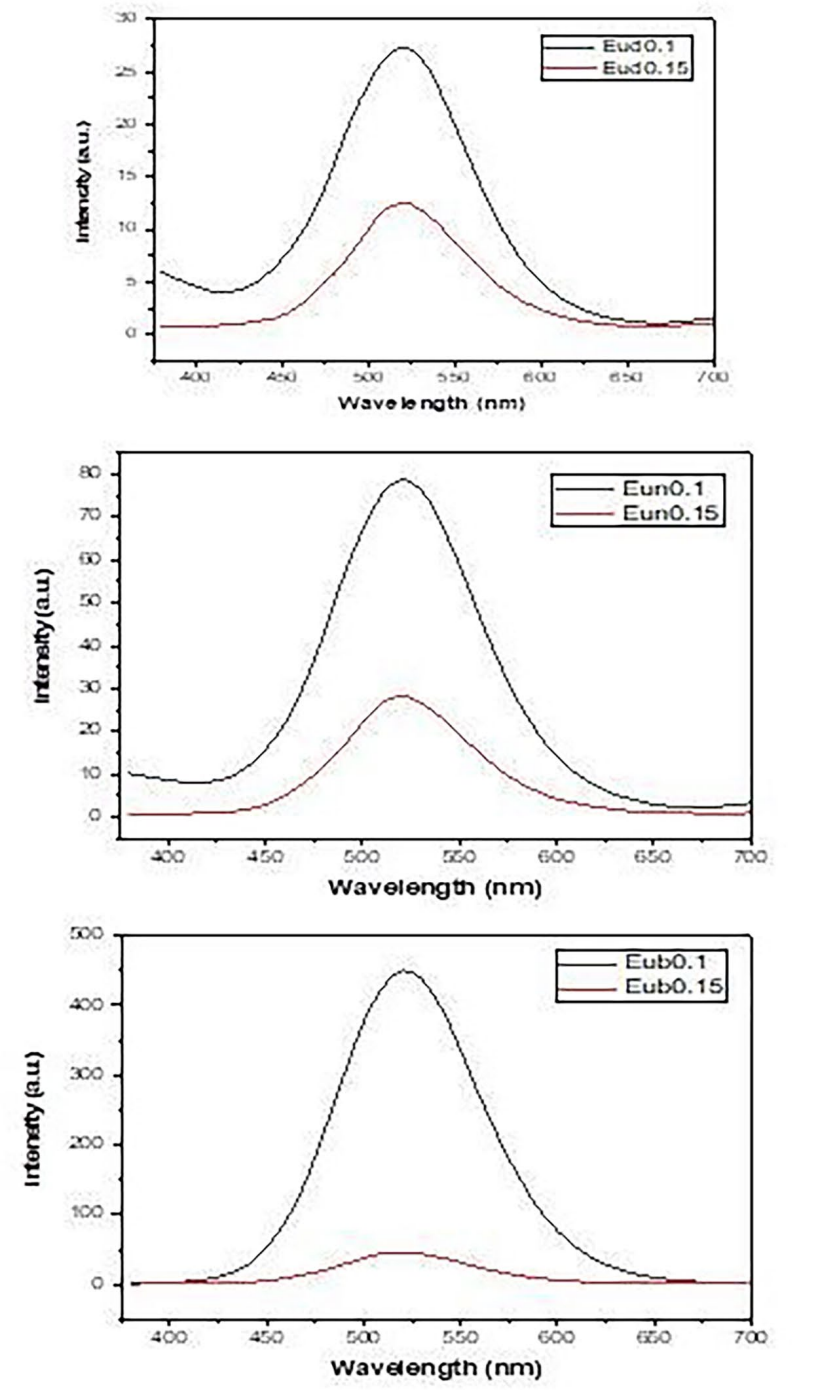
Phosphorescence spectra of samples fired under active carbon powder

Phosphorescence spectra after a 5 m sec delay time are shown in Fig. 11, which reveals the occurrence of a broad phosphorescent band at about 520 nm. The behavior of the phosphorescence intensity is nearly the same as the emission intensity, indicating that the electronic transition of Eu^2+^ (4f^6^ → 5d^1^4f^7^) dominates the phosphorescence process.

**Fig. 12:**
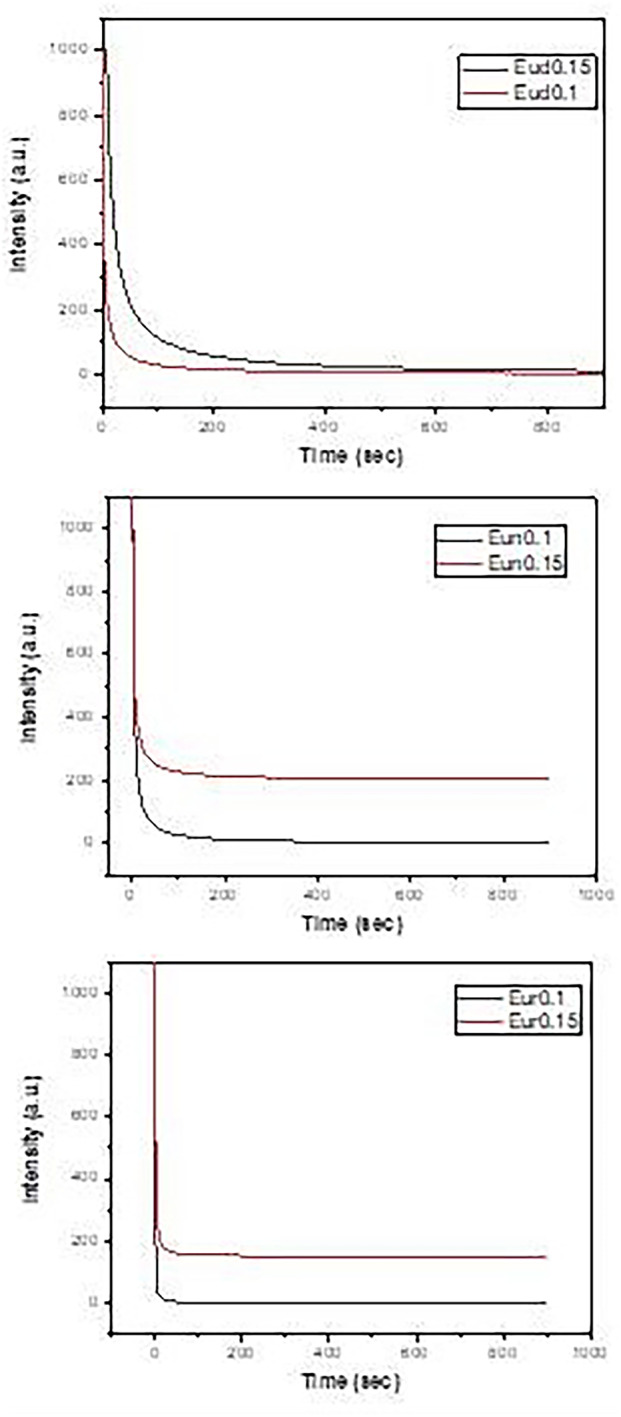
Decay curves of the prepared samples

Decay curves are shown in Fig. 12, which reveals the highest decay time (Table 2) values for samples containing Dy phosphor, then doped Nd and doped B in all concentrations.

**Table 2:** Lifetimes of phosphors samples fired under active carbon at 1250 °C

Sample	τ1	τ2	τ3	Average lifetime (sec)
Eud0.1	9.283	29.286	151.601	94.950
Eud0.15	2.383	16.164	59.674	22.464
Eun0.1	1.313	8.312	49.083	26.947
Eun0.15	3.107	21.223	67.754	21.361
Eur0.1	0.493	5.563	-	2.653
Eur0.15	0.800	4.505	32.631	12.076

There are two main assumptions to explain the afterglow phenomenon of the co-doped SrAl_2_O_4_:Eu^2+^ phosphors. The first mechanism showed that UV irradiation induces a hole-type photoconductivity and hence suggested the existence of a hole trapping [63]. Upon UV illumination, the excitation transitions of Eu^2+^ 4f^7^ → 4f^6^5d^1^ occur immediately, causing a large number of holes and electrons. The holes are transferred by the valence band and then trapped by Dy^3+^ ions to form Dy^4+^ ions. When the source of light is removed, the holes trapped by Dy^3+^ are thermally excited and transferred back to the excited state Eu^1+^ to form Eu^2+^, which leads to the long afterglow of the phosphors. This mechanism suggests that the long afterglow is mainly due to the trap levels and whether they were located at a suitable depth. It means that in a specific range of trap levels, the deeper trap depth can lead to a longer afterglow time. The second assumption proposed that the co-doped cation ions induce the formation of hole traps, accompanied by Sr^2+^ vacancies introduced according to charge compensation [64].

However, the afterglow is observed even if the co-doped cation ions are not added, which indicates that the co-doped ions are not necessarily hole traps. The hole traps are suggested to be formed by imperfections found in SrAl_2_O_4_:Eu^2+^. The Addition of the co-doped ions (Dy, Nd, B) will increase the Sr^2+^ vacancies by charge compensation. The mode of incorporation of Nd and Dy ions into the lattice is different from that of boron. Nd and Dy’s ions occupy likely Sr^2+^ ions sites, where their ionic radii are very close. On the other hand, boron ions occupy Al^3+^ ion sites and form Al-O-B bonds [65]. The present afterglow decay data showed that the samples co-doped with Dy and Nd have longer afterglow than those co-doped with boron, which is consistent with the second assumption. However, there is still the probability that the co-doped ions can act as hole traps because the afterglow process of these phosphors is a thermo-activated physical phenomenon in which charge carriers (electrons/holes) are released at well-defined temperatures characteristic of the trap depths [66]. Based on the previous discussion, the low emission intensity of SrAl_2_O_4_:Eu, Dy phosphors indicates the formation of deep hole traps by Dy^3+^ ions, which results in low emission intensity and also long afterglow time. In comparison, Nd^3+^ and B^3+^ ions form shallow hole traps, considering that Nd^3+^ ions are more efficient in the formation of deeper hole traps than B^3+^ ions.

In general, the Eud0.1, Eun0.1and Eur0.1 samples show improvement in emission, excitation, Phosphorescence and decay time behaviors may be due to RE is part of compound RESr_2_AlO_5_:Eu^2+^ (RE = Dy, Nd, B), As electron or holes may trap into a large compound which contributes to the sample’s energy-storage carrier performance [22].

It was observed that phase structure and crystallinity have a significant impact on luminescence intensity. In general, it is expected that more crystalline phosphors will be more luminescent and have fewer traps [67].

Phsosherance emission and excitation spectra of the prepared samples (Eud0.1, Eun0.1, and Eub0.1) that demonstrated long-lasting luminescent behavior, as determined by decay time results, allow it to be utilized in a variety of applications, including luminous paint, electronic instrument dial pads, writing and printing inks, automotive dials, panels, and plasma display phosphors, as well as safety indicators on emergency devices.

## Conclusion

This study successfully prepared two Phosphor materials. The first is RESr_2_AlO_5_:Eu^2+^ surrounded by SrAl_2_O_4_: Eu^2+^, RE^3+^ (RE: Dy, Nd, B). The second phosphor is Eu^2+^, RE^3+^: SrAl_2_O_4_. the novelty of firing samples in active carbon is achieved It was observed that the increase in tri-metal oxide concentration positively affected the characteristics of photoluminescence materials. This resulted in the development of materials that generate light continuously for extended periods, applicable in various areas such as oxygen sensors, glow-in-the-dark watch dial pads, and luminous paint.

## Challenge

In our future work, we will continue to develop new synthetic strategies that allow precise control of the morphology, surface, and defect chemistry of the nanomaterials, as well as an understanding of the physical mechanisms taking place in the nanoscale, aiming to propel their applicability in vivo imaging, optical data storage, or unconventional light generation, photoluminescent textiles and anti-counterfeiting patterns.

## Data Availability

The datasets generated and/or analyzed during the current study are not publicly available because they are private, but are available from the corresponding author on reasonable request.
